# Ruminal Transcriptomic Analysis of Grass-Fed and Grain-Fed Angus Beef Cattle

**DOI:** 10.1371/journal.pone.0116437

**Published:** 2015-06-19

**Authors:** Yaokun Li, José A. Carrillo, Yi Ding, YangHua He, Chunping Zhao, Linsen Zan, Jiuzhou Song

**Affiliations:** 1 College of Animal Science and Technology, Northwest A&F University, Yangling, Shaanxi, P.R. China, 712100; 2 Department of Animal & Avian Sciences, University of Maryland, College Park, MD, 20742, United States of America; Cornell University, UNITED STATES

## Abstract

Beef represents a major diet component and one of the major sources of protein in human. The beef industry in the United States is currently undergoing changes and is facing increased demands especially for natural grass-fed beef. The grass-fed beef obtained their nutrients directly from pastures, which contained limited assimilable energy but abundant amount of fiber. On the contrary, the grain-fed steers received a grain-based regime that served as an efficient source of high-digestible energy. Lately, ruminant animals have been accused to be a substantial contributor for the green house effect. Therefore, the concerns from environmentalism, animal welfare and public health have driven consumers to choose grass-fed beef. Rumen is one of the key workshops to digest forage constituting a critical step to supply enough nutrients for animals’ growth and production. We hypothesize that rumen may function differently in grass- and grain-fed regimes. The objective of this study was to find the differentially expressed genes in the ruminal wall of grass-fed and grain-fed steers, and then explore the potential biopathways. In this study, the RNA Sequencing (RNA-Seq) method was used to measure the gene expression level in the ruminal wall. The total number of reads per sample ranged from 24,697,373 to 36,714,704. The analysis detected 342 differentially expressed genes between ruminal wall samples of animals raised under different regimens. The Fisher’s exact test performed in the Ingenuity Pathway Analysis (IPA) software found 16 significant molecular networks. Additionally, 13 significantly enriched pathways were identified, most of which were related to cell development and biosynthesis. Our analysis demonstrated that most of the pathways enriched with the differentially expressed genes were related to cell development and biosynthesis. Our results provided valuable insights into the molecular mechanisms resulting in the phenotype difference between grass-fed and grain-fed cattle.

## Background

As the integral part of food production system, cattle not only make valuable contributions to the diversity of human food supply, but also play a main role in nutrient recycling and still constitute a significant work force in some countries. In the next decades, large demand for beef could be foreseeable for most developing countries and particularly for those with large populations and rapid demographic growth rate. Therefore, it is necessary for researchers to enhance the animal productivity through the application of appropriate technologies, particularly in production system and nutrient digestion [1]. Although the increment of meat production is critical for the years to come, the improvement in composition and quality of the beef is also essential. Among the beef characteristics, flavor, as the combination of taste and aroma, is one of the most important factors affecting consumer preference [2]. Nowadays, the market is more demanding for the flavor, quality and composition of beef. Additionally, with regard to the perceived divergence in nutritional quality between grass-fed and grain-fed cattle, growing consumers were interested in grass-fed beef products due to the decreasing fatty acid content [3]. It was reported that grass finished beef had higher concentration of diterpenoids and derivatives of chlorophyll, which changed the aroma and flavor of the cooked beef [4]. In addition, studies on lamb suggested that the meat from concentrate feeding was more tender and juicer than the grass-fed lambs; meanwhile, the carcass was heavier, with more fatty and less liver flavor in animals from concentrate diets [5]. The beef composition also diverged between grass-fed and grain-fed cattle. For instance, beta-carotene was the precursor of retinol (Vitamin A), a fat-soluble vitamin critical for bone growth, cell differentiation and division, and reproduction [6]. As compared to grain-fed animals, pasture-fed cattle had significantly larger amounts of beta-carotene in their muscles. Vitamin E, another fat-soluble vitamin with eight different isoforms, had powerful antioxidant activity [7]. Numerous studies have demonstrated higher concentration of vitamin E in the meat of grass-fed cattle compared with products from concentrate diets [8–10].

In the course of evolution, ruminants developed a forestomach where bacteria, fungi and protozoa disintegrated the forage under anaerobic conditions. Functionally, the reticulum and rumen constituted a unit called reticulorumen, where the ingesta mixed constantly to facilitate microbial digestion. Ruminal pillars projected the aliment into the rumen and the contraction of the walls promoted the circulation of contents in the reticulorumen. Additionally, rumination drove the decrease of size and the increase of density in the particles [11]. This series of processes were necessary to supply adequate nutrition for animals’ maintenance, growth and production. Rumen formed the lager part of the reticulorumen and served as the main site for plant material digestion and microbial fermentation.

Angus, as one of the most famous cattle breeds of the world, contributed to large proportion of beef yield, especially in America. Surrounding it, numerous valuable researches were carried on. For instance, most studies were carried on focusing on rumen microbes and its fermentation effects [12–15]. Whereas, little information about ruminal transcriptome was reported; the molecular mechanism of feed digestion and nutritional absorption remained largely unknown.

In the project, we hypothesize that rumen may function differently under grass-fed and grain-fed regimes, which result in different compositions and flavor of beef. To test it, we choose the ruminal wall tissue as our primary experiment material. The RNA sequencing (RNA-Seq) method was used to identify differentially expressed genes (DEGs) in the ruminal wall of grass-fed and grain-fed bovines. Then, based on the DEGs list, we performed a computational function analysis and found potential mechanisms contributing to the difference between the two groups.

## Materials and Methods

### Sample collection

Ruminal wall samples from two randomly chosen animals per group were obtained, totaling four samples. The animals were born, raised and maintained at the Wye Angus farm. This herd, which has been closed for almost 75 years and yielded genetically similar progenies, constitutes an excellent resource to perform transcriptomic analysis. The genetic resemblance among individuals permits us to better control the cause of variation between experimental clusters and individuals. The randomly chosen pairs of animals were part of larger sets of steers that received a particular treatment. All animals received the same diet until weaning. The grain group received conventional diet consisting of corn silage, shelled corn, soy bean and trace minerals. The grass fed steers consumed normally grazed alfalfa; during wintertime, bailage was utilized. The alfalfa has been harvested from land without any fertilizers, pesticides or other chemicals. The steers ate no animal, agricultural or industrial byproducts and never receive any type of grain. Then, the calves were randomly assigned to one diet and exclusively received that regimen until termination. Grain—fed animals reached the market weight around the age of 14 month-old, however, grass-fed steers required approximately 200 additional days to achieve the same weight. Immediately after termination at the Old Line Custom Meat Company (Baltimore, MD) a small piece of ruminal wall was excised, cleaned and preserved at -80°C for posterior processing.

### RNA extraction and sequencing

Total RNA was extracted individually (two animals per group) using Trizol (Invitrogen, Carlsbad, CA, USA) followed by DNase digestion and Qiagen RNeasy column purification (Invitrogen, Carlsbad, CA, USA), as previously described [16]. The RNA sample was dissolved in RNAse-free H_2_O; the integrity and quality of RNA were then checked by a NanoDrop 1000 spectrophotometer and by resolution on a 1.5% non-denaturing agarose gel. Each library was identified by adding 6-bp adaptors and sequenced at 50 bp/sequence read using an Illumina HiSeq 2000 sequencer, as described previously [17]. Approximately 30 million reads per sample were generated.

### Data analysis and bioinformatics

After we got the raw sequenced reads data, we checked the quality through FastQC [18], which is an online tool with the capability to report the quality profile of the reads. Then, alignment to the reference genome (Bos_taurus_UMD3.1/bosTau6) obtained from the UCSC (http://genome.ucsc.edu/) was performed employing Bowtie (Ultrafast, memory-efficient short read aligner). During this step, the first 15 bases of each read (50 bp) were trimmed to avoid low Phred quality scores, resulting in 35 bp tags. The counting of reads per gene was executed by the summarizeOverlaps function implemented in R. The identification of differentially expressed genes was achieved employing the edgeR software package and the included generalized linear model (GLM) approach, which requires a design matrix to describe the treatment conditions (grass-fed and grain-fed). In edgeR, an effective library size was computed using a scaling factor based on library sizes. The normalization was model-based and the original counts were not transformed. For variance calculation, edgeR first estimated a common dispersion for all reads and then employed a Bayesian strategy to force the tagwise variation towards the common dispersion, increasing the detection sensitivity. The threshold for calling a differentially expressed gene was false discovery rate (FDR) <0.1.

Through online software David Bioinformatics Resources 6.7, we performed the GO enrichment analysis and analyzed the biological process, cellular component and molecular function associated with the DEGs [19,20]. The enrichment of the GO terms was decided by Fisher’s exact test. Ingenuity Pathways Analysis (IPA, Ingenuity Systems, and www.ingenuity.com) was further utilized to analyze the genetic networks, molecular functions and molecular pathways enriched in the DEGs [21]. IPA, a highly convenient software application, can sanction biologists to classify the pathways, molecular networks and functions most relevant to genes of interest or experimental datasets [22–26]. Fisher’s exact test was utilized to calculate a p-value across the process of IPA analysis.

### Quantitative real-time PCR (qRT-PCR) analysis

qRT-PCR was conducted to validate and compare the expression of several randomly selected DEGs found in the RNA-Seq analysis on the iCycler iQ PCR system (Bio-Rad, Hercules, CA, USA). The template cDNA was obtained through the iScript First Strand Synthesis System Kit (Bio-Rad) for reverse transcriptase-PCR with 500 ng of total RNA. The RT-PCR reactions were performed with a QuantiTect SYBR Green PCR Kit (Qiagen, Valencia, CA, USA) according to the manufacturer’s instructions. An online primer system (http://frodo.wi.mit.edu/primer3/) was used to design the primers. Three technical replicates and two independent biological replicates were performed for each product. GAPDH was selected as the control gene [27]. All the primer sequences were listed in S1 Table.

## Results

### Alignment of sequencing reads

In total, 24,697,373 to 36,714,704 sequence reads were generated per sample. Table 1 summarized the alignment results. For the four samples, the alignment level exceeded 82%. The number of reads in the grain-fed group was greater than that in the grass-fed group; however, the alignment proportion comparison demonstrated the opposite direction (S1 Fig). Sequencing results showed that 24,616 genes could be considered for the analysis (S2 Table). Among these genes, 342 displayed significantly differential expression levels for the FDR less than 0.1 (S3 Table).

**Table 1:** Alignment of RNA-Seq Reads to the Bovine Genome.

Sample	Total reads	Total aligned reads	% Aligned
Grass1	24697373	20914897	84.68%
Grass2	28894211	24923677	86.26%
Grain1	36714704	30181834	82.21%
Grain2	35576341	29870573	83.96%

### Gene expression analysis

**Fig 1:**
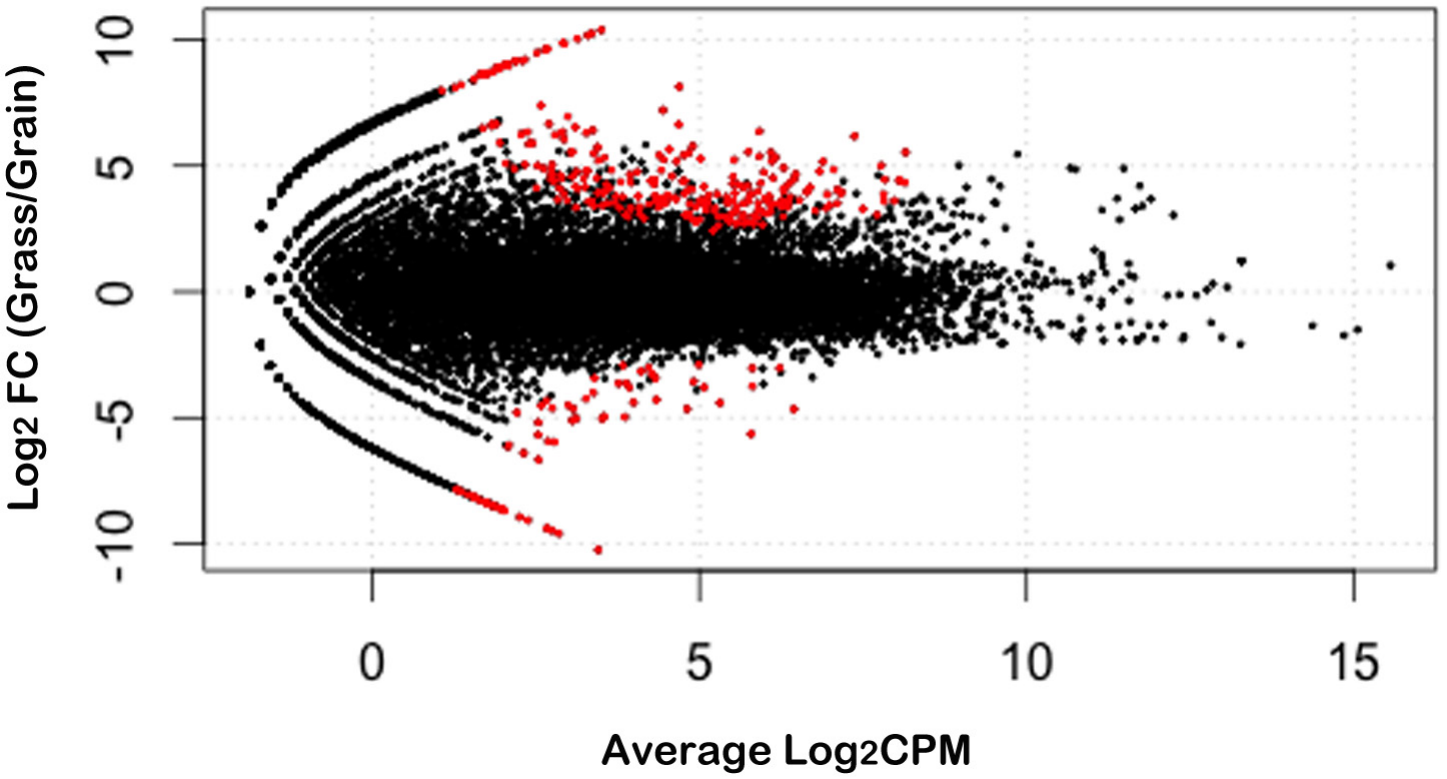
Differentially expressed rumen genes between grass-fed and grain-fed steers. MA-plot obtained from two independent biological replicates for FDR < 0.1. The red points refer to the genes with differential expression. The ordinate represents log2 fold change. CPM means counts per million.

The edgeR package implemented in R environment was applied during the statistical analysis to detect the genes with divergent expression profiles in the ruminal wall of grass-fed and grain-fed steers. The threshold criteria to call a significant difference was FDR<0.1. In total, 342 genes with distinct expression levels in both groups were distinguished following this methodology (Fig 1). From these genes, 267 were highly expressed in the ruminal wall of grass-fed bovines compared to the grain-fed group. The other 75 genes were down-regulated in grass-fed steers. The expression level of 82 genes in grass-fed steers ruminal wall was up-regulated with log_2_FC (fold-change) ≥5. The reads amount of 44 genes in grain-fed group increased significantly with the log_2_FC ≥5. The top 10 DEGs in the ruminal wall of the two groups were provided in Table 2. Among these genes, the expression levels of GALNT15, MFAP5, ADAMTS15 and RSPO3 were all higher in grain-fed group than that in grass-fed with the log_2_FC > 5. For the other 6 genes, the expression abundance was lower in grain-fed steers. The whole DEGs discovered between the two groups can be found in S3 Table.

**Table 2:** Top 10 differentially expressed genes in the ruminal wall of grass-fed and grain-fed Angus Cattle.

Ensemble Gene ID	Symbol	Log_2_ FC (grass/grain)	CPM (Grass/Grain)	FDR
ENSBTAG00000008240	GALNT15	-10.2324	87.60/60.11	2.22×10^–5^
ENSBTAG00000012584	GJB3	10.03519	45.47/78.42	2.22×10^–5^
ENSBTAG00000034848	F2RL1	9.832995	29.64/76.81	1.42×10^–4^
ENSBTAG00000013831	DSG1	10.24195	10.94/126.94	3.73×10^–4^
ENSBTAG00000000310	MFAP5	-9.58044	51.10/42.88	5.73×10^–4^
ENSBTAG00000014329	LOC512548	10.38487	5.89/145.61	7.81×10^–4^
ENSBTAG00000016857	ADAMTS15	-9.47078	45.41/41.68	8.67×10^–4^
ENSBTAG00000008121	RSPO3	-9.40866	40.54/42.89	1.14×10^–3^
ENSBTAG00000033510	MPZL2	5.518227	124.84/797.15	1.17×10^–3^
ENSBTAG00000018647	SLC2A11	5.49098	67.23/82.15	1.34×10^–3^

Note: CPM means counts per million.

### Validation of DEGs by qPCR

**Fig 2:**
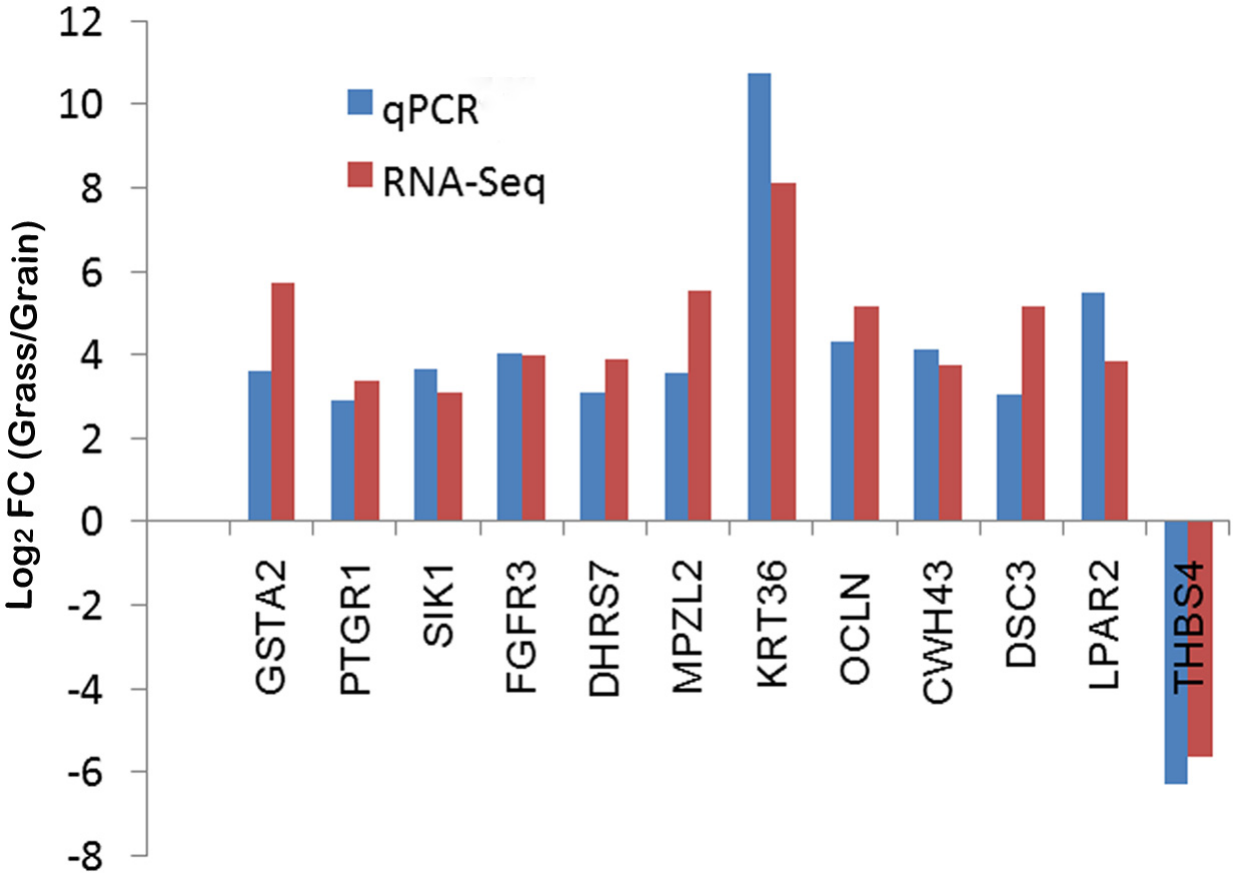
Validation of differentially expressed genes. The mean value of log_2_ (fold-change) for each group was compared in the bar chart for the 12 selected genes. qPCR data was normalized by GAPDH expression for each sample. Means of significant (FDR≤0.1) fold changes were computed for qPCR and DESeq using sample from the same 4 animals in each analysis. FC means fold-change.

Twelve DEGs were randomly selected and analyzed by qPCR as described previously [28]. The results were then compared to the same genes analyzed by edgeR (Fig 2). Among these twelve genes, all of them were in good agreement for consistency of response. For gene THBS4, the expression level in grain-fed ruminal wall was significantly higher than in grass-fed ruminal wall, and the results of qPCR and RNA-Seq suggested the same direction with almost the same log_2_FC value. For the other eleven genes, the abundance level of grass-fed steers was higher than grain-fed group. Overall, the validation of the twelve selected genes by qPCR confirmed the accuracy of RNA-Seq analysis results.

### Gene Ontology enrichment analysis

To explore the specific functional features shared by the DEGs, online software David Bioinformatics Resources 6.7 was used to perform the GO enrichment analysis. Results showed that some biological processes, cellular components and molecular functions were significantly enriched in the DEGs (Table 3). The most significant GO terms in the above three categories were: homophilic cell adhesion in biological process (P = 9.31×10^–6^), plasma membrane in cellular component (P = 2.00×10^–8^) and calcium ion binding in molecular function (P = 1.54×10^–2^). Other enriched GO terms included oxidation reduction, regulation of cell proliferation, negative regulation of cell proliferation, positive regulation of cell differentiation, cell junction, plasma membrane part, enzyme inhibitor activity and carbohydrate binding.

**Table 3:** Gene Ontology (GO) terms enriched with differentially expressed genes (GO level > 6).

GO terms	Observed*	P	FDR
***Biological process***			
GO:0007156: homophilic cell adhesion	10	9.31×10^−6^	9.40×10^−3^
GO:0016337: cell-cell adhesion	12	1.70×10^−5^	8.60×10^−3^
GO:0055114: oxidation reduction	22	1.43×10^−4^	4.73×10^−2^
GO:0007155: cell adhesion	16	2.06×10^−4^	5.10×10^−2^
GO:0022610: biological adhesion	16	2.06×10^−4^	5.10×10^−2^
GO:0042127: regulation of cell proliferation	13	2.44×10^−3^	3.90×10^−1^
GO:0008285: negative regulation of cell proliferation	8	2.60×10^−3^	3.56×10^−1^
GO:0045597: positive regulation of cell differentiation	7	3.94×10^−3^	4.35×10^−1^
GO:0006955: immune response	12	1.10×10^−2^	7.13×10^−1^
GO:0051094: positive regulation of developmental process	7	1.23×10^−2^	6.81×10^−1^
GO:0009611: response to wounding	8	2.92×10^−2^	8.12×10^−1^
GO:0006952: defense response	9	3.58×10^−2^	8.14×10^−1^
GO:0008284: positive regulation of cell proliferation	7	4.56×10^−2^	8.38×10^−1^
GO:0006811: ion transport	14	9.31×10^−2^	9.16×10^−1^
***Cellular component***			
GO:0005886: plasma membrane	51	2.00×10^−8^	2.95×10^−6^
GO:0030054: cell junction	16	8.04×10^−6^	5.95×10^−4^
GO:0005911: cell-cell junction	10	7.58×10^−5^	3.73×10^−3^
GO:0044459: plasma membrane part	30	9.27×10^−5^	3.42×10^−3^
GO:0005576: extracellular region	33	3.77×10^−4^	1.11×10^−2^
GO:0070161: anchoring junction	7	1.35×10^−3^	3.28×10^−2^
GO:0031012: extracellular matrix	10	7.87×10^−3^	1.22×10^−1^
GO:0044421: extracellular region part	17	8.73×10^−3^	1.22×10^−1^
GO:0005578: proteinaceous extracellular matrix	9	1.42×10^−2^	1.61×10^−1^
GO:0009986: cell surface	7	3.40×10^−2^	3.25×10^−1^
***Molecular function***			
GO:0005509: calcium ion binding	17	1.54×10^−2^	7.40×10^−1^
GO:0030414: peptidase inhibitor activity	7	1.72×10^−2^	6.99×10^−1^
GO:0004857: enzyme inhibitor activity	8	2.03×10^−2^	6.94×10^−1^
GO:0030246: carbohydrate binding	7	3.80×10^−2^	8.54×10^−1^

*Number of the differentially expressed genes in the category.

“GO level > 6” means that each GO term in this table contains more than 6 differentially expressed genes of the 342 genes of interest discovered in our study.

### Pathway enrichment by Ingenuity Pathway Analysis

Through Fisher’s exact test in the Ingenuity Pathway analysis (IPA) system, we then detected the genes that were involved in the canonical pathways. The 13 most significantly enriched pathways were shown in Table 4. Importantly, majority pathways were related to cell development and biosynthesis. Among them, we emphasized PXR/RXR activation, glutathione redox reactions II, LPS/IL-1 mediated inhibition of RXR function, vitamin-C transport, agranulocyte adhesion and diapedesis, estrogen biosynthesis and triacylglycerol biosynthesis. This result would provide prior knowledge to explain the difference between grass-fed and grain-feed Angus cattle.

**Table 4:** Canonical pathways enriched with differentially expressed genes by Ingenuity Pathway Analysis (IPA) (P < 0.05).

Ingenuity Canonical Pathways	Observed*	P value	FDR
Granulocyte adhesion and diapedesis	8	0.0024	0.4332
PXR/RXR activation	5	0.0025	0.2229
Glutathione redox reactions II	2	0.0035	0.2088
Ascorbate recycling (cytosolic)	2	0.0089	0.3960
LPS/IL-1 mediated inhibition of RXR function	8	0.0098	0.3519
Retinoate biosynthesis I	3	0.0118	0.3520
Glutathione-mediated detoxification	3	0.0147	0.3759
Triacylglycerol biosynthesis	3	0.0215	0.4811
Estrogen biosynthesis	3	0.0242	0.4813
Vitamin-C transport	2	0.0310	0.5549
Glutathione redox reactions I	2	0.0367	0.5972
Agranulocyte adhesion and diapedesis	6	0.0375	0.5594
CDP-diacylglycerol biosynthesis I	2	0.0459	0.6320

*Number of the differentially expressed genes in the category.

### Molecular subnetwork

**Fig 3:**
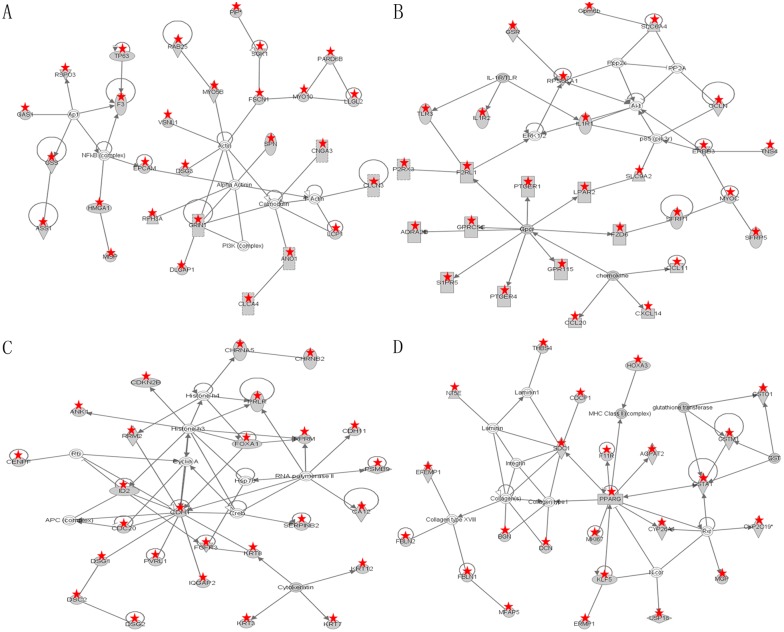
The top four molecule networks identified by Ingenuity Pathway Analysis (IPA). A: The most significant molecular network by IPA pathway analysis. B: The second most significant molecular network. C: The third most significant molecular network. D: The fourth most significant molecular network.

With additional criteria that each pathway should have at least 10 DEGs and the pathway’s score should be above 10, a total of 16 significant molecular networks were found by Fisher’s exact test in the IPA system (S4 Table). The pathway’s score was calculated by the transformation from—logP, where P is generated by Fisher’s exact test [29]. Fig 3 showed the four most significant networks. In the first network (Fig 3A), 28 DEGs were observed, and the most important functions of this network consisted of molecular transport and organ morphology. The second network, including 27 DEGs, was enriched with the function of cell-to-cell signaling and interaction, immunological disease and connective tissue disorders (Fig 3B). The third network involved 26 DEGs; the top function of this network was embryonic development, organ development and organismal development (Fig 3C). The fourth network, in which we observed 24 DEGs, was mainly related to the function of drug metabolism and gastrointestinal disease (Fig 3D).

## Discussion

Indeed, development of animal genetic improvement and breeding methodology can bring about leaner beef products [30]. Regardless of the genetic makeup, species, age, gender and geographic location, grass and grain rations of the diet can also contribute to remarkable discrepancy in the general fatty acid profile and antioxidant content in the body tissues and lipid depots [8,31,32]. And the potential changes of rumen metabolism function may have effects on the quality and quantity of protein reaching other digestive organs, such as reticulum, small intestine and large intestine; the ratio of protein from the digestion in the rumen may alter as the rumen started to digest the dry feed [33]. The disparate proportion between undegraded feed protein and bacterial protein reaching the lower gut would also lead to the shift of the quality and quantity of the protein available for absorption [33]. Therefore, our research mainly focused on the ruminal wall based on the grass-fed and grain-fed regimen to explore the underlying molecular mechanisms and biopathways.

In response to dietary changes, 60 differentially expressed proteins in sheep ruminal epithelium were detected after two days of hay-fed and concentrate-fed; 6 weeks later, only 14 proteins displayed disparate expression level [34]. By altering the dietary plane of nutrition, Aisha et al. detected 208 genes with distinct expression level in the ruminal epithelium tissue of young Holstein calves, which lead to a strong transcriptomic response [35]. In our present study, 342 DEGs were found between grass-fed and grain-fed ruminal wall of Angus cattle. Seventy-eight percent of these genes displayed significantly higher expression level in grass-fed steers. This might be explained by that the distinct feed style caused the transcriptome difference. Studies also suggested that, compared to grain-finished beef, grass-finished beef had higher concentration of beta-carotene [36], glutathione [37] and less total fat [3]. Our analysis indicated that, among the top 10 DEGs, DSG1 is related to embryonic, organ and organismal development; RSPO3 is related to abnormal morphology and organismal death. According to previous studies, the structure characterization of DSG1 encoding the pemphigus antigen has been analyzed [38]; in the absence of DSG1, the phosphorylation of the RNA polymerase II carboxy-terminal domain may be transformed, which would affect the recruitment of RNA processing machinery [39]. RSPO3 is a novel protein in the Wnt signaling pathway, which was one of the key pathways controlling cell differentiation, cell proliferation and morphogenesis [40]. Till now, there is little information about the function of these two genes, which may be the potential genetic factors that gave rise to the different carcass and growth rate between grass-fed and grain-fed cattle.

After identification of DEGs, pathway analysis was performed to better understand the biological function of the DEGs in the context of the regulatory system. Providing the information about the molecular networks and the pathways enriched in the DEGs, it becomes possible for us to explore the gene action and regulation, searching for the explanation of the underlying molecular mechanism in the discrepancy between the two groups. IPA analysis showed that the DEGs GLRX, GSTO1 were involved in the canonical pathway vitamin-C transport, which may alter beef color, lipid stability and fatty acid composition between grass-fed and grain-fed cattle [41]. In our study, molecular network analysis elucidated that there were 26 DEGs involved in the third significant network; the prior function of this network consisted of embryonic, organ and organismal development. Between grass-fed and grain-fed cattle, studies have demonstrated that the carcass composition was also different [42]. Glutathione functions as a component of the enzyme system consisting of glutathione oxidase and reductase; compared to grain-fed beef, glutathione was particularly high in grass-fed beef [3]. Our studies indicated that, between grass-fed and grain-fed cattle, the differentially expressed gene GSR was involved in the pathways glutathione redox reactions II and glutathione redox reactions I; another two DEGs GLRX and GPX7 respectively functioned in glutathione redox reactions II and glutathione redox reactions I. Accordingly, it might be intriguing to perform functional experiment of these genes on cattle to better comprehend the mechanisms causing the varied production performance.

Our results might be informative to explain the molecular mechanisms leading to the differences between grass-fed and grain-fed cattle, including the beef color, fatty acid content, vitamin concentration and carcass. However, there are still some limitations in our current work. The DEGs and the follow-up pathway/network analysis were conducted merely relying on the computational strategies; extensive experimental validation work is still needed. Thus, overexpressing and inhibiting the important differential genes in the pathways or networks could be considered for the functional validation, which can provide more supportive and valuable evidence for our findings.

## Conclusions

Through genome-wide RNA-Sequencing of the genes expressed in the ruminal wall of grass-fed and grain-fed Angus cattle, we were able to identify the genes and pathways that may affect the growth and meat quality traits of cattle. Totally, 342 DEGs were discovered between grass-fed and grain-fed cattle. Combining network and differential gene expression analysis, we detected the genes related to embryonic and organ development, organismal development and death, including DSG1 and RSPO3. According to those DEGs, a total of 16 significant molecular networks, involved in organ morphology, immunological disease, embryonic development, organ and organismal development, were found in the IPA system. Most of the pathways enriched in the DEGs were associated with cell development and biosynthesis. While expanding the scope of future studies with putative genes relevant to bovine growth and meat quality traits, our findings provided more insights into the mechanisms to enhance the productivity of animals.

## Supporting Information

S1 Fig Alignment of RNA-Seq reads to the Bovine Genome. (TIF)

S1 Table Primers used for quantitative real-time PCR validation. (XLS)

S2 Table Common genes shared by all samples. (XLS)

S3 Table Differentially expressed genes in the ruminal wall of grass-fed and grain-fed cattle at a strict false discovery rate (FDR) <0.1. (XLS)

S4 Table The 16 most significant molecular networks found by Fisher’s exact test in the IPA system. (XLS)

## References

[1] Wanapat M, Kang SC, Phesatcha K (2013) Enhancing Buffalo Production Efficiency through Rumen Manipulation and Nutrition. Buffalo Bulletin 32: 258–275.

[2] Maughan C, Tansawat R, Cornforth D, Ward R, Martini S (2012) Development of a beef flavor lexicon and its application to compare the flavor profile and consumer acceptance of rib steaks from grass- or grain-fed cattle. Meat Sci 90: 116–121. 10.1016/j.meatsci.2011.06.006 21703775

[3] Daley CA, Abbott A, Doyle PS, Nader GA, Larson S (2010) A review of fatty acid profiles and antioxidant content in grass-fed and grain-fed beef. Nutrition Journal 9. 10.1186/1475-2891-9-10 PMC2846864 20219103

[4] Elmore JS, Warren HE, Mottram DS, Scollan ND, Enser M, et al (2004) A comparison of the aroma volatiles and fatty acid compositions of grilled beef muscle from Aberdeen Angus and Holstein-Friesian steers fed diets based on silage or concentrates. Meat Sci 68: 27–33. 10.1016/j.meatsci.2004.01.010 22062004

[5] Priolo A, Micol D, Agabriel J, Prache S, Dransfield E (2002) Effect of grass or concentrate feeding systems on lamb carcass and meat quality. Meat Science 62: 179–185. 22061409 10.1016/s0309-1740(01)00244-3

[6] Scott LW, Dunn JK, Pownall HJ, Brauchi DJ, Mcmann MC, et al (1994) Effects Of Beef And Chicken Consumption on Plasma-Lipid Levels In Hypercholesterolemic Men. Archives Of Internal Medicine 154: 1261–1267. 8203993

[7] Descalzo AM, Insani EM, Biolatto A, Sancho AM, Garcia PT, et al (2005) Influence of pasture or grain-based diets supplemented with vitamin E on antioxidant/oxidative balance of Argentine beef. Meat Sci 70: 35–44. 10.1016/j.meatsci.2004.11.018 22063278

[8] De la Fuente J, Diaz MT, Alvarez I, Oliver MA, Furnols MFI, et al (2009) Fatty acid and vitamin E composition of intramuscular fat in cattle reared in different production systems. Meat Science 82: 331–337. 10.1016/j.meatsci.2009.02.002 20416720

[9] Realini CE, Duckett SK, Brito GW, Dalla Rizza M, De Mattos D (2004) Effect of pasture vs. concentrate feeding with or without antioxidants on carcass characteristics, fatty acid composition, and quality of Uruguayan beef. Meat Science 66: 567–577. 10.1016/S0309-1740(03)00160-8 22060866

[10] Yang A, Brewster MJ, Lanari MG, Tume RK (2002) Effect of vitamin E supplementation on alpha-tocopherol and beta-carotene concentrations in tissues from pasture- and grain-fed cattle. Meat Science 60: 35–40. 22063103 10.1016/s0309-1740(01)00102-4

[11] Beck M, Reuter T, Lindner S, Richert H, Hoffmann M (2013) Recording the Movement Behaviour of a Bolus in the Rumen of Cattle with a Magnetic Detector System. Biomed Tech (Berl). 10.1515/bmt-2013-4277 24042913

[12] Pinloche E, McEwan N, Marden JP, Bayourthe C, Auclair E, et al (2013) The Effects of a Probiotic Yeast on the Bacterial Diversity and Population Structure in the Rumen of Cattle. Plos One 8. 10.1371/journal.pone.0067824 PMC3699506 23844101

[13] Yang CJ, Zou CX, Liang X, Wei SJ, Li SL, et al (2013) Rumen Bacterial Diversity of Water Buffalo (Bubalus bubalis) as Influenced by Concentrate Levels. Buffalo Bulletin 32: 951–951.

[14] Wanapat M, Kang S, Khejornsart P, Wanapat S (2013) Effects of Plant Herb Combination Supplementation on Rumen Fermentation and Nutrient Digestibility in Beef Cattle. Asian-Australasian Journal Of Animal Sciences 26: 1127–1136. 10.5713/ajas.2013.13013 25049893 PMC4093230

[15] Castrillo C, Mota M, Van Laar H, Martin-Tereso J, Gimeno A, et al (2013) Effect of compound feed pelleting and die diameter on rumen fermentation in beef cattle fed high concentrate diets. Animal Feed Science And Technology 180: 34–43.

[16] Li RW, Li C (2006) Butyrate induces profound changes in gene expression related to multiple signal pathways in bovine kidney epithelial cells. BMC Genomics 7: 234 16972989 10.1186/1471-2164-7-234 PMC1592091

[17] Li RW, Schroeder SG (2012) Cytoskeleton remodeling and alterations in smooth muscle contractility in the bovine jejunum during nematode infection. Funct Integr Genomics 12: 35–44. 10.1007/s10142-011-0259-7 22203460

[18] S. A (2010) FastQC: a quality control tool for high throughput sequence data. Available: http://www.bioinformatics.babraham.ac.uk/projects/fastqc. Accessed 12 December 2014.

[19] Young MD, Wakefield MJ, Smyth GK, Oshlack A (2010) Gene ontology analysis for RNA-seq: accounting for selection bias. Genome Biol 11: R14 10.1186/gb-2010-11-2-r14 20132535 PMC2872874

[20] Huang DW, Sherman BT, Tan Q, Kir J, Liu D, et al (2007) DAVID Bioinformatics Resources: expanded annotation database and novel algorithms to better extract biology from large gene lists. Nucleic Acids Research 35: W169–W175. 17576678 10.1093/nar/gkm415 PMC1933169

[21] Kramer A, Green J, Pollard J, Tugendreich S (2014) Causal analysis approaches in Ingenuity Pathway Analysis. Bioinformatics 30: 523–530. 10.1093/bioinformatics/btt703 24336805 PMC3928520

[22] Su YQ, Sugiura K, Woo Y, Wigglesworth K, Kamdar S, et al (2007) Selective degradation of transcripts during meiotic maturation of mouse oocytes. Dev Biol 302: 104–117. 17022963 10.1016/j.ydbio.2006.09.008 PMC1847322

[23] Abdel-Aziz HO, Takasaki I, Tabuchi Y, Nomoto K, Murai Y, et al (2007) High-density oligonucleotide microarrays and functional network analysis reveal extended lung carcinogenesis pathway maps and multiple interacting genes in NNK [4-(methylnitrosamino)-1-(3-pyridyle)-1-butanone] induced CD1 mouse lung tumor. J Cancer Res Clin Oncol 133: 107–115. 16977459 10.1007/s00432-006-0149-x PMC12160851

[24] Pospisil P, Iyer LK, Adelstein SJ, Kassis AI (2006) A combined approach to data mining of textual and structured data to identify cancer-related targets. BMC Bioinformatics 7: 354 16857057 10.1186/1471-2105-7-354 PMC1555615

[25] Mayburd AL, Martlinez A, Sackett D, Liu H, Shih J, et al (2006) Ingenuity network-assisted transcription profiling: Identification of a new pharmacologic mechanism for MK886. Clin Cancer Res 12: 1820–1827. 16551867 10.1158/1078-0432.CCR-05-2149

[26] Calvano SE, Xiao W, Richards DR, Felciano RM, Baker HV, et al (2005) A network-based analysis of systemic inflammation in humans. Nature 437: 1032–1037. 16136080 10.1038/nature03985

[27] Mitra A, Luo J, Zhang H, Cui K, Zhao K, et al (2012) Marek's disease virus infection induces widespread differential chromatin marks in inbred chicken lines. BMC Genomics 13: 557 10.1186/1471-2164-13-557 23072359 PMC3505159

[28] Merrick BA, Auerbach SS, Stockton PS, Foley JF, Malarkey DE, et al (2012) Testing an Aflatoxin B1 Gene Signature in Rat Archival Tissues. Chemical Research In Toxicology 25: 1132–1144. 10.1021/tx3000945 22545673 PMC3358548

[29] Jia PL, Kao CF, Kuo PH, Zhao ZM (2011) A comprehensive network and pathway analysis of candidate genes in major depressive disorder. Bmc Systems Biology 5. 10.1186/1752-0509-5-S3-S12 PMC3287567 22784618

[30] Higgs JD (2000) The changing nature of red meat: 20 years of improving nutritional quality. Trends In Food Science & Technology 11: 85–95.

[31] De Smet S, Raes K, Demeyer D (2004) Meat fatty acid composition as affected by fatness and genetic factors: a review. Animal Research 53: 81–98.

[32] Garcia PT, Pensel NA, Sancho AM, Latimori NJ, Kloster AM, et al (2008) Beef lipids in relation to animal breed and nutrition in Argentina. Meat Science 79: 500–508. 10.1016/j.meatsci.2007.10.019 22062910

[33] Quigley JD 3rd, Schwab CG, Hylton WE (1985) Development of rumen function in calves: nature of protein reaching the abomasum. J Dairy Sci 68: 694–702. 3989088 10.3168/jds.S0022-0302(85)80875-4

[34] Bondzio A, Gabler C, Badewien-Rentzsch B, Schulze P, Martens H, et al (2011) Identification of differentially expressed proteins in ruminal epithelium in response to a concentrate-supplemented diet. Am J Physiol Gastrointest Liver Physiol 301: G260–268. 10.1152/ajpgi.00304.2010 21566014

[35] Naeem A, Drackley JK, Lanier JS, Everts RE, Rodriguez-Zas SL, et al (2014) Ruminal epithelium transcriptome dynamics in response to plane of nutrition and age in young Holstein calves. Functional & Integrative Genomics 14: 261–273. 24318765 10.1007/s10142-013-0351-2

[36] Descalzo AM, Insani EM, Biolatto A, Sancho AM, Garcia PT, et al (2005) Influence of pasture or grain-based diets supplemented with vitamin E on antioxidant/oxidative balance of Argentine beef. Meat Science 70: 35–44. 10.1016/j.meatsci.2004.11.018 22063278

[37] Descalzo AM, Rossetti L, Grigioni G, Irurueta M, Sancho AM, et al (2007) Antioxidant status and odour profile in fresh beef from pasture or grain-fed cattle. Meat Science 75: 299–307. 10.1016/j.meatsci.2006.07.015 22063662

[38] Puttagunta S, Mathur M, Cowin P (1994) Structure Of Dsg1, the Bovine Desmosomal Cadherin Gene Encoding the Pemphigus Foliaceus Antigen—Evidence Of Polymorphism. Journal Of Biological Chemistry 269: 1949–1955. 8294446

[39] Muratani M, Kung C, Shokat KM, Tansey WR (2005) The F box protein Dsg1/Mdm30 is a transcriptional coactivator that stimulates Gal4 turnover and cotranscriptional mRNA processing. Cell 120: 887–899. 15797387 10.1016/j.cell.2004.12.025

[40] Ohkawara B, Glinka A, Niehrs C (2011) Rspo3 Binds Syndecan 4 and Induces Wnt/PCP Signaling via Clathrin-Mediated Endocytosis to Promote Morphogenesis. Developmental Cell 20: 303–314. 10.1016/j.devcel.2011.01.006 21397842

[41] Realini CE, Duckett SK, Windham WR (2004) Effect of vitamin C addition to ground beef from grass-fed or grain-fed sources on color and lipid stability, and prediction of fatty acid composition by near-infrared reflectance analysis. Meat Science 68: 35–43. 10.1016/j.meatsci.2004.02.002 22062005

[42] Priyanto R, Johnson ER, Taylor DG (1993) Prediction Of Carcass Composition In Heavy-Weight Grass-Fed And Grain-Fed Beef-Cattle. Animal Production 57: 65–72. 8454553

