# Correction: Ruminal Transcriptomic Analysis of Grass-Fed and Grain-Fed Angus Beef Cattle

**DOI:** 10.1371/journal.pone.0134067

**Published:** 2015-07-21

**Authors:** Yaokun Li, José A. Carrillo, Yi Ding, YangHua He, Chunping Zhao, Linsen Zan, Jiuzhou Song

Supporting Information S1 Fig appears incorrectly in the published article. Please view the correct S1 Fig here.

## Supporting Information

S1 Fig Alignment of RNA-Seq reads to the Bovine Genome. (TIF)
