# Supplementary figures and images for: Ruminal Transcriptomic Analysis of Grass-Fed and Grain-Fed Angus Beef Cattle
Source: PLoS One. 2015 Jun 19;10(6):e0116437. doi: 10.1371/journal.pone.0116437 (PMC4475051; doi:10.1371/journal.pone.0116437)

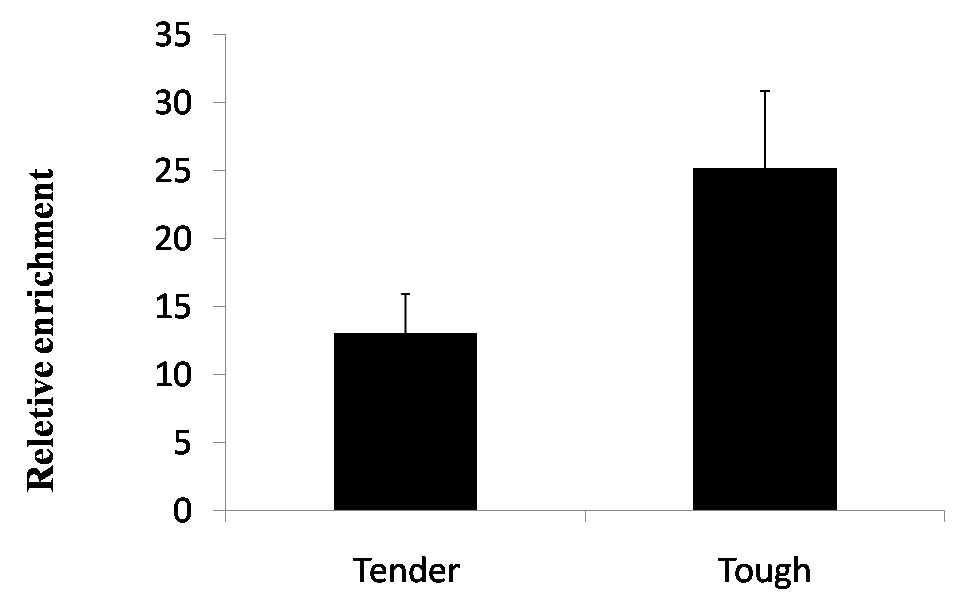

Supplement: S1 Fig — (TIF) [file pone.0116437.s001.tif]
