# Supplementary figures and images for: Correction: Ruminal Transcriptomic Analysis of Grass-Fed and Grain-Fed Angus Beef Cattle
Source: PLoS One. 2015 Jul 21;10(7):e0134067. doi: 10.1371/journal.pone.0134067 (PMC4510587; doi:10.1371/journal.pone.0134067)

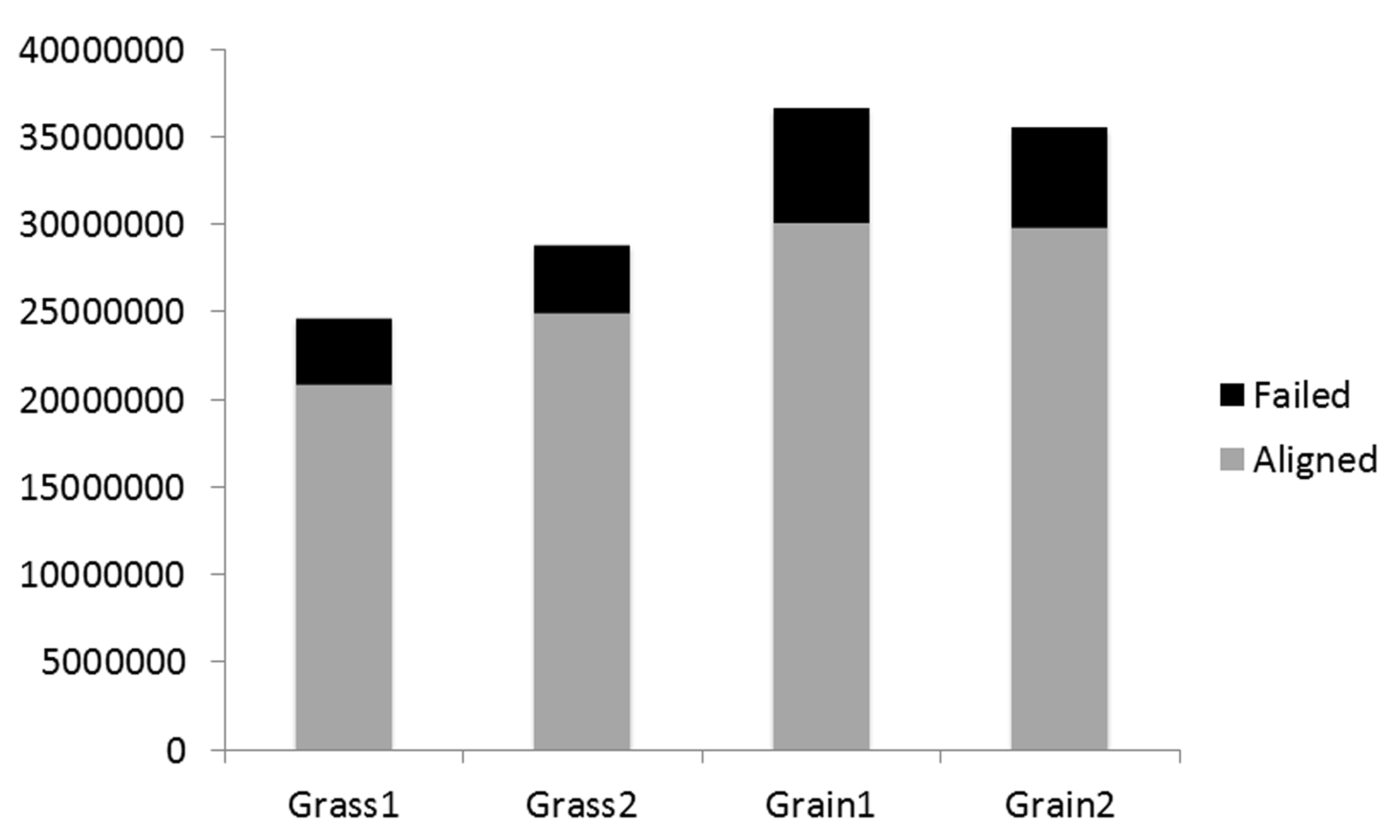

Supplement: S1 Fig — (TIF) [file pone.0134067.s001.tif]
